# Female‐biased gape and body‐size dimorphism in the New World watersnakes (tribe: Thamnophiini) oppose predictions from Rensch's rule

**DOI:** 10.1002/ece3.5492

**Published:** 2019-08-09

**Authors:** Frank T. Burbrink, India Futterman

**Affiliations:** ^1^ Department of Herpetology The American Museum of Natural History New York NY USA

**Keywords:** gartersnakes, Rensch's rule, sexual‐size dimorphism, trait shifts, watersnakes

## Abstract

**Abstract:**

Sexual‐size dimorphism (SSD) is ubiquitous across animals and often biased in the direction of larger females in snakes and other ectothermic organisms. To understand how SSD evolves across species, Rensch's rule predicts that in taxa where males are larger, SSD increases with body size. In contrast, where females are larger, SSD decreases with body size. While this rule holds for many taxa, it may be ambiguous for others, particularly ectothermic vertebrates. Importantly, this rule suggests that the outcomes of SSD over phylogenetic time scales depend on the direction of dimorphism predicated on the difference in reproductive efforts between males and females. Here, we examine SSD in the context of Rensch's rule in Thamnophiini, the gartersnakes and watersnakes, a prominent group that in many areas comprises the majority of the North American snake biota. Using a dated phylogeny, measurements of gape, body, and tail size, we show that these snakes do not follow Rensch's rule, but rather female‐biased SSD increases with body size. We in turn find that this allometry is most pronounced with gape and is correlated with both neonate and litter size, suggesting that acquiring prey of increased size may be directly related to fecundity selection. These changes in SSD are not constrained to any particular clade; we find no evidence of phylogenetic shifts in those traits showing SSD. We suggest several ways forward to better understand the anatomical units of selection for SSD and modularity.

**Open Research Badges:**

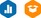

This article has been awarded Open Data and Open Materials Badges. All materials and data are publicly accessible via the Open Science Framework at https://doi.org/10.5061/dryad.3pn57h0.

## 1 INTRODUCTION

Sexual dimorphism often accounts for most of the morphological variation seen within a sexually reproducing species (Darwin, 1871; Fairbairn, Blanckenhorn, & Székely, 2007). Two main themes account for dimorphism both predicated on the reproductive differences between males and females and the production of phenotypes at unique optima (Fairbairn, 2013; Rohner, Teder, Esperk, Lüpold, & Blanckenhorn, 2018). Dimorphism may be the result of intersexual selection, such as when females prefer unique attributes among males, or intrasexual selection. Intrasexual competition among males can result in massively different body sizes and unique structures not found in females (Fairbairn, 1997; Fairbairn et al., 2007). Similarly, female‐dominated size differences between sexes also occur when selection drives reproductive output in females (Ralls, 1976). Importantly, over phylogenetic time scales, sexual dimorphism can follow a few trends depending on which sex is larger, known as Rensch's rule (Rensch, 1950, 1959).

As one of the latest contributors to the modern synthesis, Bernard Rensch addressed problems related to understanding diversification and commented on general trends in sexual‐size dimorphism (SSD) at taxonomic scales above the population (Rensch, 1950, 1959). Rensch stated that in taxa where males are larger, SSD increases with body size. In contrast, where females are larger, SSD decreases with body size. This suggests in part that selection on SSD at the level of the species is thus magnified in effect at larger body sizes for males with weaker effects on females (Chown & Gaston, 2010; Webb & Freckleton, 2007). This can, of course, be driven by sexual selection favoring larger males; however, in the case of female‐biased SSD, this pattern may be the result of female competition for mates, differences in age between the sexes, or as in many cases, due to fecundity selection, where greater energy is expended to produce offspring in greater number or more frequently (Cox, Skelly, & John‐Alder, 2003; Fairbairn, 1997; Liao, Zeng, Zhou, & Jehle, 2013; Preziosi & Fairbairn, 2000; Remeš & Székely, 2010). Finally, correlational selection may tie selective increases in one sex with that of another sex (Liao, Liu, & Merilä, 2015).

While Rensch's rule is well supported in male‐biased SSD, it is often not for the reverse female‐biased SSD (Webb & Freckleton, 2007). In several studies, the trajectory of female‐biased SSD is reflective of Rensch's rule for males, where the degree of SSD increases with body size (Liao, 2013; Liao & Chen, 2012; Lu, Zhou, Zhao, & Liao, 2014; Webb & Freckleton, 2007). While more rarely studied, selection in support of Rensch's rule may not act on body size alone, but may affect different parts of anatomy independently. Modularity across organismal anatomy in the context of sexual dimorphism has been examined previously (Emlen, Hunt, & Simmons, 2005; Preziosi & Fairbairn, 2000; Taylor et al., 2017), but generally understudied with respect to testing Rensch's rule. However, size and shape of cranial sexual dimorphism in New World opossums has been shown to not follow Rensch's rule given body size (Astúa de Moraes, 2010).

In addition, it is often unknown whether Rensch's rule is being driven by threshold changes in specific clades; species with significant SSD at larger body sizes may not be evenly distributed across the phylogeny but rather constrained to particular clades (Baker & Wilkinson, 2001; Ceballos, Adams, Iverson, & Valenzuela, 2013). This would then suggest that SSD might not change continuously over the phylogeny with body size, but rather occur in leaps associated with significant shifts in morphospace within clades, which itself may be associated with adaptive radiation within particular niches.

For the most part, ectotherms, and particularly snakes, show more female‐biased SSD than endotherms (Cox, Butler, & John‐Alder, 2007). In those cases, Rensch's rule may or may not be supported (Webb & Freckleton, 2007). Specifically, snakes often show female‐biased SSD (Shine, 1994) which can be associated with consuming larger prey and therefore increased fecundity or transition to viviparity (Daltry, Wuster, & Thorpe, 1998; Shine, 1994, 2003; Stuart‐Fox, 2009). For some snake clades, combat among males, mate‐searching, and sperm competition are associated with male‐biased SSD (Bonnet, Shine, Naulleau, & Vacher‐Vallas, 1998). At deeper phylogenetic levels, a few studies on some colubrid and elapid snakes have suggested that where female‐biased SSD is known, positive interspecific allometry is weak and supports Rensch's rule (Abouheif & Fairbairn, 1997; Fairbairn, 1997; Webb & Freckleton, 2007). Unfortunately, detailed comparative phylogenetic studies on snake SSD and Rensch's rule for well‐known snake groups with comprehensive sampling have not been examined.

Some limited studies have suggested that Rensch's rule is supported in snakes showing female‐biased SSD (Abouheif & Fairbairn, 1997; Fairbairn, 1997; Webb & Freckleton, 2007). For snakes, width and size of the head and gape are correlated with prey size (Vincent, Dang, Herrel, & Kley, 2006), may show differences among sex (Shine, 1978), and are often associated with differences in prey type. This is most prominent where females consume larger or different types of prey (Mushinsky, Hebrard, & Vodopich, 1982; Shine, 1991; White & Kolb, 1974) and may be associated with enhanced fecundity (Cox et al., 2007; Seigel & Ford, 1987; Shine, 2000). Sexually selected traits may reveal positive allometries relative to body size (Eberhard et al., 2018) and modularity via selection (Goswami, Smaers, Soligo, & Polly, 2014; Klingenberg, 2014; Olson & Miller, 1958), but it is unclear whether SSD with respect to Rensch's rule shows the outcomes of independent selection on these two features.

Here, we examine gape, body, and tail size in New World gartersnakes, watersnakes, and related taxa (Thamnophiini) to understand whether female‐biased sexual‐size dimorphism reflects Rensch's rule and shows unique allometry across body and tail size and gape. We also determine if increases in body size are related to increased fecundity by associating body size with litter and neonate size. Using a phylogenetic approach, we determine if SSD is concentrated in specific clades of watersnakes or if they are randomly distributed across the tree. These snakes have been well‐studied ecologically, behaviorally, morphologically, and phylogenetically and are a prominent feature of the snake fauna of North America (Burbrink, Chen, Myers, Brandley, & Pyron, 2012; Gibbons & Dorcas, 2004; Guo et al., 2012; McVay, Flores‐Villela, & Carstens, 2015; Rossman, Ford, & Seigel, 1996), though the evolution of SSD across this group is unknown. Our results help understand if increases or decreases in SSD follow Rensch's rule, are associated with reproduction, and are reveal shared changes in particular groups of these snakes.

## 2 METHODS

### 2.1 Data

We examined only adults of 1,535 specimens among 49 thamnophiine taxa (81.7% species, remaining taxa were unavailable in sufficient number to properly estimate error around measurements; Data S1–[Link], [Link], [Link]). Where hemipenes were not fixed externally, each specimen was sexed by making a small incision into the ventral side of the tail to identify the presence or absence of hemipenes. Snout–vent length (SVL), tail length (TL), head length (HL), and head width (HW) were measured to the nearest millimeter. For each species, we attempted to measure equal numbers of males and females of up to 20 samples for each sex. We also collected data from the literature on maximum litter and neonate size (Data S1–[Link], [Link], [Link]). Both HL and HW were transformed into a standard gape index by converting these measurements into the area of an ellipse (King, 2002). Because the formula would require the measurement of HW and HL to each be taken from the origin of the ellipse, we divided each of these measurements by 2 to account for the distance from the origin: π × (HL/2) × (HW/2). As discussed by King (2002) and Miller and Mushinsky (1990), this index is useful for estimating maximum gape and therefore suggests that HL and HW each contribute uniquely to estimating this index. To understand if these head measurements uniquely contribute to estimating gape index, rather than just assuming a single measure is a useful proxy for gape, we took raw measurements and regressed HL and HW by sex through the origin using standardized major axis regression (SMA) to determine if these deviate from isometry by rejecting a slope of 1.0 using the R package “smatr” (Warton, Duursma, Falster, & Taskinen, 2012). Therefore, even if these measurements were correlated, rate changes over the sizes of Thamnophiini could suggest that they contribute uniquely to estimating gape and therefore neither variable should be used separately as a measure of this index.

### 2.2 Testing Rensch's rule

We first log‐transformed all traits and determined if they showed significant phylogenetic signal using Blomberg's *K* test to understand if relatives resemble each other under Brownian motion. We used the function phylosig in the R package “phytools” (Revell, 2012) with the standard 1,000 simulations for the random test to yield enough power to determine significance. We removed phylogenetic nonindependence using phylogenetic independent contrasts (PIC) in the R package (R Core Team, 2018) “Ape” (Paradis, Claude, & Strimmer, 2004). We used the most recent dated species tree of Thamnophiini (Data S2) from McVay et al. (2015) to account for phylogenetic independence. To examine relationships between traits for testing SSD, we used the model II reduced/standardized major axis regression, reviewed in Smith (2009), which was used to study allometry with Rensch's rule in Wu, Jiang, Huang, and Feng (2018) in the R package “smatr” (Warton et al., 2012). We compare our SMA results to ordinary least squares (OLS) given the more frequent usage of the latter, but note that given symmetry in error estimation of variables from both sexes and the lack of a single direction of inference (e.g., male sizes predicting female sizes or vice versa) suggests that SMA is the more appropriate choice. Using PIC‐transformed variables, we regressed traits through the origin (Garland, Harvey, & Ives, 1992; Legendre & Desdevises, 2009), tested for a significant relationship, and assessed if traits were changing allometrically if slopes significantly deviated from 1.0, thus supporting Rensch's rule.

We used SMA to regress female SVL on male SVL to determine if female size changes allometrically relative to males. The way we have designed these tests, placing female trait dominance on the axis of ordinates, indicated that slopes significantly >1.0 showed that female‐biased dimorphism increased as female body size increased, whereas slopes significantly <1.0 supported male‐biased dimorphism. Slopes at 1.0, suggested no bias in sexual‐size dimorphism associated with increases in male or female body size. Similarly, we regressed the difference in female and male gape on the difference in female and male SVL, female gape on male gape, and female TL on male TL. We determined if gape allometry was significantly different from SVL allometry by seeing if gape slopes were significantly different from SVL using SMA regressions. To determine if any detected SSD allometry in favor of females was also related to litter size, we regressed litter size to female SVL. We also regressed neonate size on female SVL to understand if increased litters were also associated with increased neonate size, which would suggest that litter sizes do not increase at the expense of neonate body size.

### 2.3 Phylogenetic trait shifts

These continuous traits (gape, SVL, and TL by sex, and differences between genders) were fit to three standard models of evolution, accounting for completely neutral evolution in Brownian motion (BM), accommodating selection and drift in Ornstein–Uhlenbeck (OU), and rapid early and rapid morphological changes in early bursts (EB; Butler & King, 2004; Felsenstein, 1985; Harmon et al., 2010) using the R package “geiger” (Harmon, Weir, Brock, Glor, & Challenger, 2008). Because any of these models of trait evolution may not be an appropriate candidate for these data, we assessed model adequacy using the R program “Arbutus” (Pennell, FitzJohn, Cornwell, & Harmon, 2015). Here, we fit each model (BM, OU, and EB) to each of the traits using maximum likelihood and compared observed fits to simulations under each model (*n* = 100 simulations, a standard number of replicates for providing statistical power for rejection with these tests) using six standard test statistics that summarize violations to models and have well‐defined statistical properties, which include: (1) *M*_sig_—the mean of squared contrasts, which measures the overall rate and provides estimates if rates are over‐ or underestimated, (2) *C*_var_—the coefficient of variation of the absolute value of contrasts, where if observed values are greater than simulated estimates then rate heterogeneity is not properly estimated, (3) *S*_var_—slope of the regressed absolute value of the contrasts against expected variables, used to understand if contrasts are smaller or larger than expected given branch lengths, (4) *S*_asr_—slope of the regressed absolute value of the contrasts against the ancestral state estimated for a corresponding node, which determines if there is variation in rate relative to the value of a trait, (5) *S*_hgt_—the slope of the regressed absolute value of the contrasts against node depth, which provides estimates of variation relative to time, (6) *D*_cdf_—the D‐stat from the Kolmogorov–Smirnov test estimated by comparing the distribution of contrasts to that of a standardized normal distribution where the mean is 0 and the standard deviation is equal to the root of the mean of squared contrasts, which estimates deviations from normality (Pennell et al., 2015). We used Akaike information criteria weights considering sample size (wAICc) to determine statistical support for all models.

We examined if traits associated with allometric SSD correspond with phylogenetic shifts in these traits. Shifts in these traits across the phylogeny would suggest that SSD may be concentrated in particular clades and not necessarily occurring gradually across the phylogeny. To explore traits space in relation to phylogeny, we graphed the difference female–male gape and SVL across tree space using the function *phylomorphospace* in the R package “phytools” (Revell, 2012). We used the R package “PhylogeneticEM” (Bastide, Ané, Robin, & Mariadassou, 2018) which implements a maximum‐likelihood method to automatically determine if shifts in traits happened and where putative shifts occurred on the phylogeny of Thamnophiini. We used the scalar Ornstein–Uhlenbeck model (scOU) model in the function PhyloEM(), Kmax (maximum number of shifts) set to 10, which accounted for a very large number of expected changes for a tree with only 47 internal nodes, and the number of alpha values on the grid (nbr_alpha) set to 10. We note that higher values of alpha provide a finer grid for searching with added computational time, though our results remain constant by increasing alpha by intervals of 20 up to 100. We ran this five times to ensure consistency among results. We chose the number of best shifts by estimating the mean of Gaussian vector among 10 predicted number of possible shifts using the “LINselect” method described in Bastide et al. (2018), which uses a penalized‐likelihood approach to find the appropriate number of shifts (BGHml).

## 3 RESULTS

**Figure 1:**
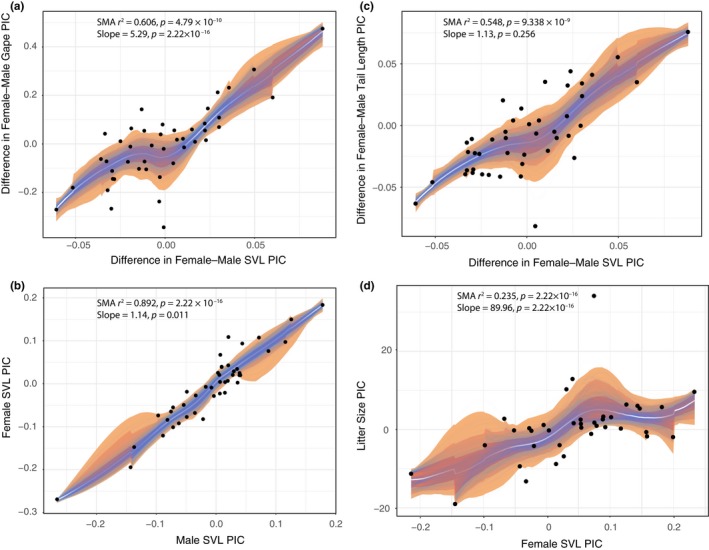
Bootstrapped regressions (light blue) and standard deviation (*SD*) for traits corrected by phylogenetically independent contrasts (PIC): (a) differences in gape by sex and snout–vent length (SVL), (b) female SVL and male SVL, and (c) difference in tail length (TL) and (d) litter size by female SVL. Statistics on each panel show on the first row standard major axis (SMA) regression coefficient of determination (*r* ^2^) and significance (*p*), and on the second line, slope and significant allometry (significance from slope = 1) are displayed

Both HL and TL measured from Thamnophiini were correlated (*ρ* = 0.98; *p *= 2.2 × 10^−16^) but each trait uniquely contributed to gape given that SMA regressions of HL and HW showed allometry with significant deviance from a slope of 1.0 by sex (*r* ^2^ = 0.99; *p* = 2.2 × 10^−16^). All traits (SVL, TL, and gape) were transformed by PIC, and each was regressed between males and females using SMA showed significance (*r* ^2^ = 0.892–0.916; *p* = 2.2 × 10^−16^; Figure 1) and supported allometry (*r* ^2^ = 0.379–0.6.23 and tests of isometry rejected at *p* = .019–7.61 × 10^−6^). Similar results for all tests were found using OLS (all other results in the text refer to SMA results), but with SVL not significantly rejecting isometry (Table S1). Difference in gape regressed against difference in SVL between females and males was significant and allometric (*r* ^2^ = 0.892; *p* = 2.22 × 10^–16^). However, difference in female and male TL regressed on difference in SVL was not significantly allometric (*r* ^2^ = 0.175; *p* = 2.56 × 10^−1^). The slope of gape difference versus TL difference on SVL was much larger for the former (gape = 5.292, TL = 1.127) and also significantly different (SMA *r* ^2^ = 0.963; *p* = 2.22 × 10^–16^). Both clutch size and neonate size regressed against SVL were significant, *r* ^2^ = 0.235 (*p* = 2.22 × 10^–16^) and *r* ^2^ = 0.281 (*p* = .001), respectively, and showed allometry (*p* = .001–.002) (Table S1).

**Figure 2:**
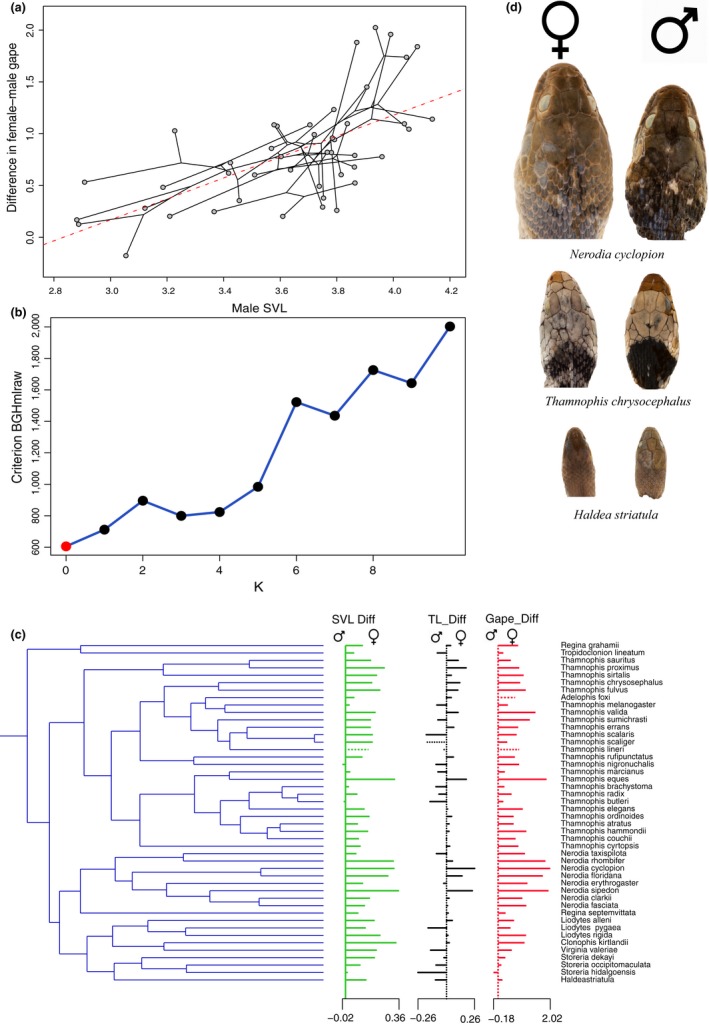
Phylogeny, trait space, and morphological shifts: (a) phylomorphospace representing the phylogeny of Thamnophiini plotted against difference in gape by sex on male snout–vent length, (b) number of shifts (*k*) in traits (difference in SVL, TL, and Gape by sex) given BGHmlraw criteria, (c) timed phylogeny of Thamnophiini showing range in trait differences between males and females and demonstrating no shifts on the phylogeny, and (d) photos showing head‐size dimorphism in three species of thamnophiines ordered from large to small taxa top to bottom, where female to male SVL ratios are approximately ~1.0

None of the trait evolution models investigated were conclusively supported given that AIC model weights (wAICc) only varied from 52% to 71% (Table S2). Brownian motion models had the highest weights on the most traits (*n* = 4), followed by OU (*n* = 3 for all trait‐difference models), and then EB (*n* = 2). Additionally, using model adequacy tests, most of the six test statistics were not significantly different (two‐tailed test) from the simulations for each of the three models. This suggested that any of the three models was an adequate fit for these trait data (Table S3). For instance, over all tests across all traits, only 4% of the statistics were significant and never a majority for any particular trait. We detected no trait shifts across Thamnophiini using the scOU model (Figure 2). All five separate runs produced the same results and selected the same model; raw BGHml model selection for number of shifts against their penalized likelihoods suggested 0 shits at 605.70 was preferred when compared to increasingly higher values after 1 shift (711.84).

## 4 DISCUSSION

In contrast to expectation and previous studies on body size, we show that gape, SVL, and TL associated with body size do not follow Rensch's rule in the watersnakes and that female‐biased SSD shows the same trend predicted for male‐biased SSD, where SSD increases with increased body sizes. Importantly, when examining the difference in female–male gape and tail size against female–male SVL, gape differences increase rapidly in comparison with difference in tail length, suggesting that larger gapes evolve independently than other size‐based traits, particularly at larger body sizes (Figure 1a,b). For these snakes, this follows if head size is predictive of consuming increased prey sizes, which as we show translates into increased fecundity.

Our results show that even though gape, SVL, and TL are each correlated between males and females showing that trends of increasing size hold for both sexes, females clearly develop much larger gapes at larger sizes (Figure 1a,b,c). These gape sizes are sexually dimorphic, likely occur at birth, and are therefore susceptible to changes driven by natural selection; a previous study on body size and head morphology on members of *Thamnophis*, *Storeria*, and *Nerodia* suggested that sexual dimorphism in these traits was genetically and not environmentally determined (King, Bittner, Queral‐Regil, & Cline, 1999). As with many snakes, garter and watersnakes species often show ontogenetic, geographic, and ecological variation in prey type and size (Gibbons & Dorcas, 2004; Rossman et al., 1996). For many species though, prey size is usually correlated with gape or body size, with females taking larger or different types of prey than males (Greene, Dixon, Mueller, Whiting, & Thornton, 1994; Mushinsky et al., 1982; Rossman et al., 1996; Shine, 1991, 1993; White & Kolb, 1974). In contrast, in smaller to medium‐sized taxa, prey‐size differences between males and females may not be as pronounced (Manjarrez, Contreras‐Garduño, & Janczur, 2014). It is worth noting that while overall size of prey increases with gape, larger species will often drop smaller prey items from their diets, which is most pronounced in fish‐eating snakes (Arnold, 1993; Godley, McDiarmid, & Rojas, 1984; Plummer & Goy, 1984) that also happen to be the largest species in Thamnophiini.

When male–male competition, finding mates, or epigamic selection does not drive SSD (Shine, 1993), it is possible that adaptation to distinct niches by sex is selected (Slatkin, 1984). For all Thamnophiini, however, there are no recorded instances of intraspecific female or male combat (Shine, 1994). One result of selection into distinct niches is that SSD in gape evolved in response to competitive displacement (Shine, 1991), though we note that this would indicate that males and females compete for limited resources (Shine, 1986), something generally not noticed in snakes, and would predict that gape and SVL should not be well correlated. More likely, gape and body size are the result of sexually selected dimorphism known to yield differences in habitat or prey size between the sexes allowing more efficient feeding (Shine, 1986, 1993). Sexual dimorphism in head size is associated with differences in prey size for many unrelated clades of snakes (Meik, Setser, Mocino‐Deloya, & Lawing, 2012; Shine, 1991, 1994). This in turn may ultimately be driven by fecundity selection, described previously within some species of watersnakes (Semlitsch & Gibbons, 1982). Here, we show that increases in body sizes are associated with increased litter sizes (Figure 1d) and, importantly, this is also associated with increased neonate size. Both sizes of clutch and neonates increase allometrically with female body size. Therefore, reproductive output is not constrained by mass; overall neonate size does not remain constant with changing litter sizes. While it is possible for selection to increase fecundity by increasing reproductive frequency (Bull & Shine, 1979), this has not been recorded in these snakes, where reproduction takes place only one time per year (Gibbons & Dorcas, 2004; Rossman et al., 1996). For Thamnophiini, the standard outcome of fecundity selection demonstrates that selection for larger female gapes and larger or longer body sizes translates into increased numbers of offspring (Braña & Brana, 1996; Darwin, 1871; Olsson, Shine, Wapstra, Ujvari, & Madsen, 2002; Valdecantos, Lobo, Perotti, Moreno Azócar, & Cruz, 2019). Likely, sexual dimorphism in Thamnophiini is the result of sexual selection on gape and diet for enhanced energy expenditure needed to increased fecundity and neonate size.

In spite of a strict interpretation of Rensch's rule, we have established tight links among evolution of SSD, interspecific allometry in key traits, and increased fecundity. While this pattern is apparent in New World natricines, it is unknown if female‐biased SSD follows a male‐biased SSD pattern in other snakes families, though females are generally larger in most clades of snakes (Shine, 1991). In those snake taxa with male‐biased SSD, it is also unclear if they follow Rensch's rule in instances with known male competition. Further comparative phylogenetic work is therefore needed to assess the degree of SSD with body size given behavior and fecundity across most snake subfamilies and families.

For Thamnophiini, we show no major shifts in SVL, TL, and gape across phylogeny (Figure 2). This indicates that the trend here, where female‐biased SSD follows male‐biased SSD for Rensch's rule, is not being driven by outlier clades showing extreme traits shifts. Some clades, like the large watersnakes, *Nerodia*, do show some of the most extreme differences in these traits between sexes and less well‐fit models (*k* = 1, 2; Figure 2) can recover this clade as having a phylogenetic shift in SSD. Similar to some members of *Thamnophis*, *Nerodia* is composed of generally larger and more aquatic snakes, has known interspecific dietary differences in prey size, and females of at least some species prefer larger fishes (Greene et al., 1994; Mushinsky et al., 1982; Rossman et al., 1996; Shine, 1991, 1993; White & Kolb, 1974).

Slopes are different among the traits examined here, with gape showing the most extreme allometry (Figure 1) suggesting some independence in the evolution of SSD among these features. From a functional context, this is not unexpected given that unique parts of the cranium, body, and tail show distinct modularity (Klaczko, Sherratt, & Setz, 2016; Klingenberg, 2008; Polly, Head, & Cohn, 2001). Given that the skull itself is modular and yet integrated for feeding, it is unclear which cranial elements are selected to increase gape and if these elements become uniformly larger in species showing greater SSD.

To better understand how SSD has evolved in snakes, future work should consider a more detailed appraisal of morphology. Clearly computed tomography scanning (CT and micro‐CT) efforts could identify exactly what structures or muscles are being selected to generate head shape differences and how these change ontogenetically. Variation in these traits in turn could be addressed in a larger phylogenetic context to better understand how changes or even reversals in SSD trends are associated with intersexual ecologies and behaviors over the snake tree of life. Finally, integrating sources of geographic variation may be useful to determine how patterns of SSD are manifested over the landscape given Berghman's clines and Rensch's rule (Blanckenhorn, Stillwell, Young, Fox, & Ashton, 2006).

## CONFLICT OF INTEREST

None declared.

## AUTHOR CONTRIBUTIONS

FTB and IF both conceived of the study designed the data collection protocol and collected data. FTB performed all statistical analyses, generated figures and wrote the original draft of the manuscript. Both FTB and IF edited the final manuscript and approved the work.

## Data Availability

All morphological and trait data as well as additional statistical results listed at Supporting Information are available via Dryad https://doi.org/10.5061/dryad.3pn57h0.
