# Supplementary material for: Female‐biased gape and body‐size dimorphism in the New World watersnakes (tribe: Thamnophiini) oppose predictions from Rensch's rule
Source: Ecol Evol. 2019 Aug 9;9(17):9624–33. doi: 10.1002/ece3.5492 (PMC6745821; doi:10.1002/ece3.5492)
Supplement: Supplementary file 5 [file ECE3-9-9624-s005.docx]

Supporting Information Table S1 SMA Statistics. Results of Standard Major Axis Tests (SMA) and ordinary least squares(OLS) for each comparison showing the r^2^, slope, upper and lower limits of those slopes, and significance. Following, we show significance for allometry (slope is not equal to 1.0)

| **SMA_Tests** | r2 | slope | lower_limit_slope | upper_limit_slope | significance | slope_sig_zero_test_Stat_slope=1 | slope_sig_zero_test_P |
| --- | --- | --- | --- | --- | --- | --- | --- |
| PIC Gape_difference_size_difference_between_Females_males | 0.606 | 5.292 | 4.359 | 6.425 | 4.79-10 | 0.971 | 2.22E-16 |
| PIC_TL_difference_size_difference_between_Females_males | 0.548 | 1.127 | 0.915 | 1.386 | 9.338-09 | 0.175 | 2.56E-01 |
| Slope_Test_from_TL=1.127_on PIC Gape_difference_size_difference_between_Females_males | 0.607 | 5.292 | 4.358 | 6.426 | 4.79E-10 | 0.963 | 2.22E-16 |
| PIC_Female_Male_SVL | 0.892 | 1.144 | 1.034 | 1.265 | 2.22E+16 | 0.379 | 0.0109 |
| PIC_Female_Male_Gape | 0.898 | 1.286 | 1.16 | 1.42 | 2.22E-16 | 0.623 | 7.608E-06 |
| PIC_Female_Male_Tail_Length | 0.916 | 1.16 | 1.061 | 1.20 | 2.22E-06 | 0.455 | 1.91E-3 |
| PIC_Clutch_Female_Size | 0.235 | 89.96 | 67.494 | 119.907 | 2.22E-16 | 0.999 | 2.22E-16 |
| PIC_Neonate_Female_SVL | 0.281 | 81.966 | 35.554 | 128.377 | 0.001 | 0.0999 | 0.001 |
| **OLS_Tests** |  |  |  |  |  |  |  |
| PIC Gape_difference_size_difference_between_Females_males | 0.606 | 4.121 | 3.088 | 5.155 | 4.79e-10 | 0.685 | 3.683e-7 |
| PIC_TL_difference_size_difference_between_Females_males | 0.548 | 0.833 | 0.598 | 1.069 | 9.338e-9 | -0.214 | 0.167 |
| Slope_Test_from_TL=1.127_on PIC Gape_difference_size_difference_between_Females_males | 0.606 | 4.122 | 3.088 | 5.155 | 4.791e-6 | 0.669 | 8.289e-7 |
| PIC_Female_Male_SVL | 0.892 | 1.080 | 0.965 | 1.196 | 2.22e-16 | 0.209 | 0.1731 |
| PIC_Female_Male_Gape | 0.898 | 1.219 | 1.091 | 1.347 | 2.22e-16 | 0.471 | 0.001 |
| PIC_Female_Male_Tail_Length | 0.916 | 1.111 | 1.006 | 1.216 | 2.22e-16 | 0.314 | 0.040 |
| PIC_Clutch_Female_Size | 0.235 | 43.631 | 17.426 | 69.838 | 0.002 | 0.476 | 0.002 |
| PIC_Neonate_Female_SVL | 0.281 | 154.56 | 114.974 | 207.79 | 128.377 | 0.526 | 0.001 |
