# Supplementary material for: Female‐biased gape and body‐size dimorphism in the New World watersnakes (tribe: Thamnophiini) oppose predictions from Rensch's rule
Source: Ecol Evol. 2019 Aug 9;9(17):9624–33. doi: 10.1002/ece3.5492 (PMC6745821; doi:10.1002/ece3.5492)
Supplement: Supplementary file 6 [file ECE3-9-9624-s006.docx]

Supporting Information Table S2 Trait Model Evolution. AICc for three models of evolution, BM (Brownian Motion), OU (Ornstein-Uhlenbeck) and EB (Early Burst) for traits (AICc weights in parentheses).

| **Trait_Evolution_Models** | BM_AICc | OU_AICc | EB_AICc |
| --- | --- | --- | --- |
| Male_SVL | 4.033816 (0.53) | 6.326175 (0.17) | 5.194286 (0.30) |
| Male_Gape | 137.000899 (0.59) | 139.300551 (0.19) | 138.91285 (0.22) |
| Male_TL | 38.672711 (0.36) | 40.972363 (0.12) | 37.987304 (0.52) |
| Female_SVL | 16.41657 (0.60) | 18.708931 (0.19) | 18.485777 (0.21) |
| Female_Gape | 159.623586 (0.55) | 161.337871 (0.23) | 161.497227 (0.22) |
| Female_TL | 25.340525 (0.30) | 27.640176 (0.09) | 23.904669 (0.61) |
| Female_Male_Gape_Diff | 68.436373 (0.35) | 67.558536 (0.54) | 70.736081 (0.11) |
| Female_Male_SVL_Diff | -81.779759 (0.22) | -84.173581 (0.71) | -79.480056 (0.07) |
| Female_Male_TL_Diff | -68.014354 (0.28) | -69.635416 (0.63) | -65.714651 (0.09) |
