# Supplementary material for: Female‐biased gape and body‐size dimorphism in the New World watersnakes (tribe: Thamnophiini) oppose predictions from Rensch's rule
Source: Ecol Evol. 2019 Aug 9;9(17):9624–33. doi: 10.1002/ece3.5492 (PMC6745821; doi:10.1002/ece3.5492)
Supplement: Supplementary file 7 [file ECE3-9-9624-s007.docx]

Supporting Information Table S3 Model Adequacy Tests. Results of model adequacy tests using “Arbutus” showing in the first column the trait by sex or gender difference in trait. Row names indicate test statistics described in Pennell et al. (2015).

| Arbutus_Model_Adequacy | m.sig | c.var | s.var | s.asr | s.hgt | d.cdf |
| --- | --- | --- | --- | --- | --- | --- |
| Female_SVL_BM | 0.87128713 | 0.05940594 | 0.43564356 | 0.37623762 | 0.97029703 | 0.91089109 |
| Female_TL_BM | 0.8118812 | 0.1584158 | 0.7722772 | 0.0990099 | 0.0990099 | 0.3168317 |
| Female_Gape_BM | 0.9306931 | 0.2772277 | 0.3168317 | 0.5940594 | 0.6930693 | 0.970297 |
| Male_SVL_BM | 0.65346535 | 0.01980198 | 0.33663366 | 0.4950495 | 0.65346535 | 0.63366337 |
| Male_TL_BM | 0.79207921 | 0 | 0.03960396 | 0 | 0.79207921 | 0.47524752 |
| Male_Gape_BM | 0.79207921 | 0.01980198 | 0.17821782 | 0.71287129 | 0.79207921 | 0.91089109 |
| SVL_Female_Male_Diff_BM | 0.8514851 | 0.9108911 | 0.9306931 | 0.9108911 | 0.1386139 | 0.4752475 |
| TL_Female_Male_Diff_BM | 0.9108911 | 0.5346535 | 0.8712871 | 0.9306931 | 0.1782178 | 0.1980198 |
| Gape_Female_Male_Diff_BM | 0.8910891 | 0.1386139 | 0.2376238 | 0.2178218 | 0 | 0.6138614 |
| Female_SVL_OU | 0.89108911 | 0.03960396 | 0.55445545 | 0.43564356 | 0.93069307 | 0.83168317 |
| Female_TL_OU | 0.87128713 | 0.23762376 | 0.57425743 | 0.15841584 | 0.05940594 | 0.35643564 |
| Female_Gape_OU | 0.6930693 | 0.1782178 | 0.2772277 | 0.6930693 | 0.950495 | 0.6138614 |
| Male_SVL_OU | 0.71287129 | 0.01980198 | 0.31683168 | 0.33663366 | 0.3960396 | 0.47524752 |
| Male_TL_OU | 0.97029703 | 0 | 0.03960396 | 0.13861386 | 0.89108911 | 0.43564356 |
| Male_Gape_OU | 0.9306931 | 0 | 0.1386139 | 0.8118812 | 0.8910891 | 0.7524752 |
| SVL_Female_Male_Diff_OU | 0.8514851 | 0.5148515 | 0.8910891 | 0.4752475 | 0.3564356 | 0.6732673 |
| TL_Female_Male_Diff_OU | 0.69306931 | 0.31683168 | 0.4950495 | 0.93069307 | 0.47524752 | 0.05940594 |
| Gape_Female_Male_Diff_OU | 0.7128713 | 0.1188119 | 0.8514851 | 0.3168317 | 0.3960396 | 0.950495 |
| Female_SVL_EB | 0.83168317 | 0.03960396 | 0.43564356 | 0.35643564 | 0.77227723 | 0.81188119 |
| Female_TL_EB | 0.8712871 | 0.3366337 | 0.8316832 | 0.0990099 | 0.3762376 | 0.2178218 |
| Female_Gape_EB | 0.6930693 | 0.1782178 | 0.2772277 | 0.6930693 | 0.950495 | 0.6138614 |
| Male_SVL_EB | 0.63366337 | 0.03960396 | 0.33663366 | 0.21782178 | 0.95049505 | 0.51485149 |
| Male_TL_EB | 0.85148515 | 0 | 0.03960396 | 0.03960396 | 0.89108911 | 0.53465347 |
| Male_Gape_EB | 0.8910891 | 0 | 0.2178218 | 0.5742574 | 0.8712871 | 0.7524752 |
| SVL_Female_Male_Diff_EB | 0.85148515 | 0.95049505 | 0.81188119 | 0.77227723 | 0.07920792 | 0.25742574 |
| TL_Female_Male_Diff_EB | 0.7722772 | 0.4752475 | 0.7524752 | 0.6732673 | 0.3366337 | 0.1188119 |
| Gape_Female_Male_Diff_EB | 0.93069307 | 0.07920792 | 0.11881188 | 0.11881188 | 0.01980198 | 0.83168317 |
